# Exact Solutions for Torsion and Warping of Axial-Loaded Beam-Columns Based on Matrix Stiffness Method

**DOI:** 10.3390/nano12030538

**Published:** 2022-02-04

**Authors:** Wen-Hao Pan, Chuan-Hao Zhao, Yuan Tian, Kai-Qi Lin

**Affiliations:** 1 College of Civil Engineering and Architecture, Zhejiang University, Hangzhou 310058, China; pan_wh@zju.edu.cn; 2 Center for Balance Architecture, Zhejiang University, Hangzhou 310028, China; 3 Architectural Design and Research Institute of Zhejiang University Co., Ltd., Hangzhou 310028, China; 4 Department of Civil Engineering, Tsinghua University, Beijing 100084, China; yuantian_10@163.com; 5 College of Civil Engineering, Fuzhou University, Fuzhou 350000, China; linkq@fzu.edu.cn

**Keywords:** matrix stiffness method, element stiffness matrix, torsion, warping, equilibrium analysis, elastic buckling analysis

## Abstract

The typically-used element torsional stiffness *GJ*/*L* (where *G* is the shear modulus, *J* the St. Venant torsion constant, and *L* the element length) may severely underestimate the torsional stiffness of thin-walled nanostructural members, due to neglecting element warping deformations. In order to investigate the exact element torsional stiffness considering warping deformations, this paper presents a matrix stiffness method for the torsion and warping analysis of beam-columns. The equilibrium analysis of an axial-loaded torsion member is conducted, and the torsion-warping problem is solved based on a general solution of the established governing differential equation for the angle of twist. A dimensionless factor is defined to consider the effect of axial force and St. Venant torsion. The exact element stiffness matrix governing the relationship between the element-end torsion/warping deformations (angle and rate of twist) and the corresponding stress resultants (torque and bimoment) is derived based on a matrix formulation. Based on the matrix stiffness method, the exact element torsional stiffness considering the interaction of torsion and warping is derived for three typical element-end warping conditions. Then, the exact element second-order stiffness matrix of three-dimensional beam-columns is further assembled. Some classical torsion-warping problems are analyzed to demonstrate the established matrix stiffness method.

## 1. Introduction

In recent decades, research on nanomaterials (i.e., materials with internal structure of nanoscale dimension) has made great progress and is widely used in scientific research and industrial production. Some formations of oxides, metals, ceramics, and other substances have been discovered. These nanomaterials obey the fundamental laws of the classical physics governing the macroworld [1]. Nanomaterials such as graphene may provide many enhanced properties including high strength, stiffness, and light weight [2]. Researchers also utilize nanomaterials in structures to improve the mechanical behavior and other performance [3].

**Figure 1:**
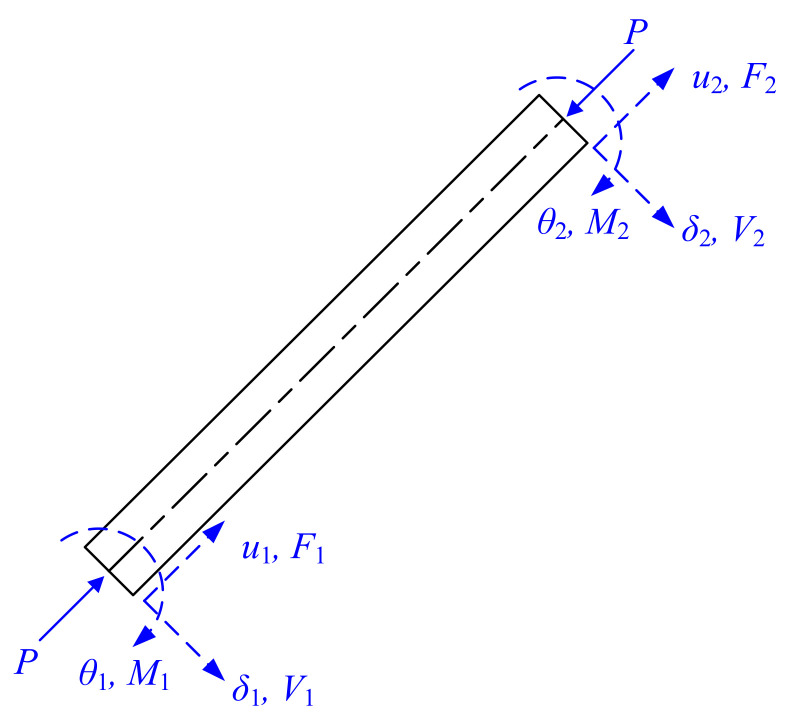
Axial-loaded beam–column with element-end displacements and forces.

Although a number of computer programs such as Abaqus, OpenSees, Ansys, and MSC.Marc are readily available for the structural analysis of thin-walled nanostructural members, approaches to obtain the exact solutions [4,5,6,7,8,9,10] in closed form are helpful in many situations. The matrix stiffness method (MSM) has been found to be a suitable and systematic method for such purposes [11,12,13,14,15,16,17,18,19,20]. The basic idea of the matrix stiffness method is to establish the equilibrium relationship between the element-end displacements $\Delta ={\left(u_{1},\;\delta_{1},\;\theta_{1},\;u_{2},\;\delta_{2},\;\theta_{2}\right)}^{T}$ and the element-end forces $F_{ext}={\left(F_{1},\;V_{1},\;M_{1},\;F_{2},\;V_{2},\;M_{2}\right)}^{T}$ of a beam-column element (where *u_i_*, *d_i_*, and *q_i_* are element-end axial displacement, translational displacement, and rotation angle, respectively; *F_i_*, *V_i_*, and *M_i_* are element-end axial load, shear force, and bending moment, respectively, as shown in Figure 1) as

(1) $$\left[K_{e}\right]\Delta =F_{ext}$$

where [***K*_e_**] is the element stiffness matrix for flexural-axial problems.

For first-order (e.g., [21,22]) and second-order (e.g., [10,13,14,15,16,19]) analysis (i.e., without and with considering the geometric nonlinearity), the element stiffness matrix of a beam-column element can be formulated as Equations (2) and (3), respectively.

(2) $$\left[K_{e,1\mathrm{st}-\mathrm{ord}}\right]=\left(\begin{array}{cccccc} \frac{EA}{L} & & & -\frac{EA}{L} & & \\ & 12\frac{EI}{L^{3}} & 6\frac{EI}{L^{2}} & & -12\frac{EI}{L^{3}} & 6\frac{EI}{L^{2}}\\ & & 4\frac{EI}{L} & & -6\frac{EI}{L^{2}} & 2\frac{EI}{L}\\ & & & \frac{EA}{L} & & \\ & & & & 12\frac{EI}{L^{3}} & -6\frac{EI}{L^{2}}\\ \mathrm{sym}. & & & & & 4\frac{EI}{L} \end{array}\right)$$

(3) $$\left[K_{e,2\mathrm{nd}-\mathrm{ord}}\right]=\left(\begin{array}{cccccc} \frac{EA}{L} & & & -\frac{EA}{L} & & \\ & T_{c}(\lambda )\frac{EI}{L^{3}} & Q_{c}(\lambda )\frac{EI}{L^{2}} & & -T_{c}(\lambda )\frac{EI}{L^{3}} & Q_{c}(\lambda )\frac{EI}{L^{2}}\\ & & S_{c}(\lambda )\frac{EI}{L} & & -Q_{c}(\lambda )\frac{EI}{L^{2}} & C_{c}(\lambda )\frac{EI}{L}\\ & & & \frac{EA}{L} & & \\ & & & & T_{c}(\lambda )\frac{EI}{L^{3}} & -Q_{c}(\lambda )\frac{EI}{L^{2}}\\ \mathrm{sym}. & & & & & S_{c}(\lambda )\frac{EI}{L} \end{array}\right).$$

where *E* denotes the elastic modulus; *A* denotes the cross-sectional area; *I* denotes the cross-sectional moment of inertia; *L* denotes the element length; and *T*_c_, *Q*_c_, *S*_c_, and *C*_c_ are coefficients in the element stiffness matrix as functions of factor *λ*, formulated as Equation (4); *λ* (in a flexural-axial problem) denotes a dimensionless factor for the axial compression force *P*_c_, defined as Equation (5).

(4) $$\begin{array}{l} T_{c}(\lambda )=\lambda^{3}\sin\lambda /\phi_{c}(\lambda )\\ Q_{c}(\lambda )=\lambda^{2}\left(1-\cos\lambda \right)/\phi_{c}(\lambda )\\ S_{c}(\lambda )=\lambda \left(\sin\lambda -\lambda \cos\lambda \right)/\phi_{c}(\lambda )\\ C_{c}(\lambda )=\lambda \left(\lambda -\sin\lambda \right)/\phi_{c}(\lambda )\\ \phi_{c}(\lambda )=2-2\cos\lambda -\lambda \sin\lambda \end{array}$$

(5) $$\lambda =\sqrt{\frac{P_{c}L^{2}}{EI}}=\pi \sqrt{\frac{P_{c}}{P_{E}}}\;$$

Using the element stiffness matrix, many different types of analyses can be conducted, including the traditional analysis for the element deformations and internal forces (e.g., [21,22]), as well as elastic buckling and second-order stability analyses (e.g., [15,16,17,18]).

**Figure 2:**
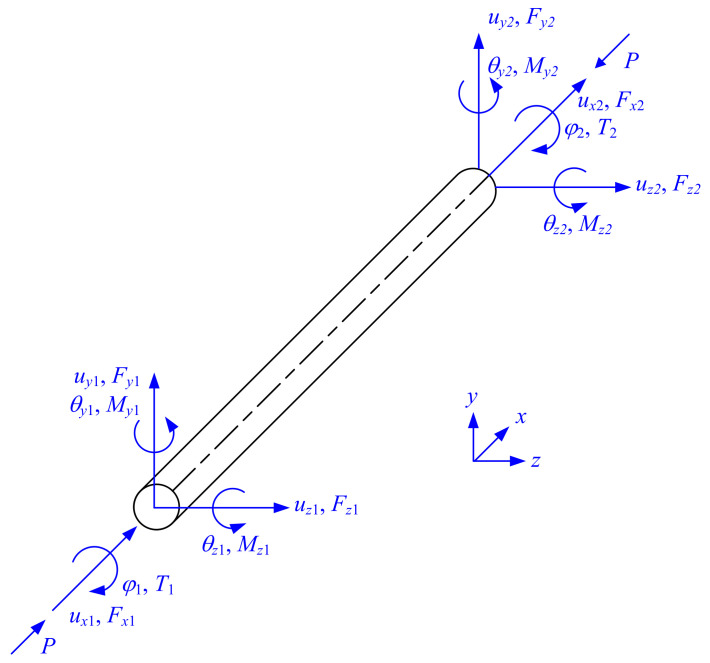
Element-end displacements and forces of 3D beam elements for traditional matrix stiffness method.

The matrix stiffness method can also be used for three-dimensional analysis of beam-columns. For second-order analysis, Ekhande [23] presented an exact element stiffness matrix associated with the 12 element-end deformations/rotation angles of 3D beam-columns as Equation (6). The matrix has been used to conduct stability analysis of 3D structures [19].

(6) $$\left(\begin{array}{cccccccccccc} \frac{EA}{L} & & & & & & -\frac{EA}{L} & & & & & \\ & T_{c}\left(\lambda_{z}\right)\frac{EI_{zz}}{L^{3}} & & & & Q_{c}\left(\lambda_{z}\right)\frac{EI_{zz}}{L^{2}} & & -T_{c}\left(\lambda_{z}\right)\frac{EI_{zz}}{L^{3}} & & & & Q_{c}\left(\lambda_{z}\right)\frac{EI_{zz}}{L^{2}}\\ & & T_{c}\left(\lambda_{y}\right)\frac{EI_{yy}}{L^{3}} & & -Q_{c}\left(\lambda_{y}\right)\frac{EI_{yy}}{L^{2}} & & & & -T_{c}\left(\lambda_{y}\right)\frac{EI_{yy}}{L^{3}} & & -Q_{c}\left(\lambda_{y}\right)\frac{EI_{yy}}{L^{2}} & \\ & & & \frac{GJ}{L} & & & & & & -\frac{GJ}{L} & & \\ & & & & S_{c}\left(\lambda_{y}\right)\frac{EI_{yy}}{L} & & & & Q_{c}\left(\lambda_{y}\right)\frac{EI_{yy}}{L^{2}} & & C_{c}\left(\lambda_{y}\right)\frac{EI_{yy}}{L} & \\ & & & & & S_{c}\left(\lambda_{z}\right)\frac{EI_{zz}}{L} & & -Q_{c}\left(\lambda_{z}\right)\frac{EI_{zz}}{L^{2}} & & & & C_{c}\left(\lambda_{z}\right)\frac{EI_{zz}}{L}\\ & & & & & & \frac{EA}{L} & & & & & \\ & & & & & & & T_{c}\left(\lambda_{z}\right)\frac{EI_{zz}}{L^{3}} & & & & -Q_{c}\left(\lambda_{z}\right)\frac{EI_{zz}}{L^{2}}\\ & & & & & & & & T_{c}\left(\lambda_{y}\right)\frac{EI_{yy}}{L^{3}} & & Q_{c}\left(\lambda_{y}\right)\frac{EI_{yy}}{L^{2}} & \\ & & & & & & & & & \frac{GJ}{L} & & \\ & \mathrm{sym}. & & & & & & & & & S_{c}\left(\lambda_{y}\right)\frac{EI_{yy}}{L} & \\ & & & & & & & & & & & S_{c}\left(\lambda_{z}\right)\frac{EI_{zz}}{L} \end{array}\right)\left(\begin{array}{c} u_{x1}\\ u_{y1}\\ u_{z1}\\ \varphi_{1}\\ \theta_{y1}\\ \theta_{z1}\\ u_{x2}\\ u_{y2}\\ u_{z2}\\ \varphi_{2}\\ \theta_{y2}\\ \theta_{z2} \end{array}\right)=\left(\begin{array}{c} F_{x1}\\ F_{y1}\\ F_{z1}\\ T_{1}\\ M_{y1}\\ M_{z1}\\ F_{x2}\\ F_{y2}\\ F_{z2}\\ T_{2}\\ M_{y2}\\ M_{z2} \end{array}\right)$$

where *G* denotes the shear modulus; *J* denotes the St. Venant torsion constant; *I_yy_* and *I_zz_* denote the cross-sectional moments of inertia about *y*-axis and *z*-axis, respectively; *λ_y_* and *λ_z_* denote axial force factors associated with the *y*-axis bending and *z*-axis bending, respectively; $u_{yi}$ and $u_{zi}$ denote element-end translational displacements in y-direction and z-direction, respectively; $\varphi_{i}$ denotes the element-end angle of twist; $\theta_{yi}$ and $\theta_{zi}$ denote element-end rotation angles in the X-Z plane and X-Y plane, respectively; $F_{yi}$ and $F_{zi}$ denote element-end loads in the y-direction and z-direction, respectively; $T_{i}$ denotes the element-end torsional moment; and $M_{yi}$ and $M_{zi}$ denote element-end bending moments about the y-axis and z-axis, respectively, as shown in Figure 2.

However, these researchers neglected the interaction between torsion and warping and the axial force effect in torsional analysis. They directly used *GJ*/*L* as torsional stiffness in engineering practices, which may severely underestimate the torsional stiffness of thin-walled nanostructural members without considering warping deformations. In order to present the exact element stiffness matrix for the second-order analysis of three-dimensional beam-columns considering torsion and warping, this paper investigates the interaction of the axial force, torque, and bimoment of torsion members. The exact element torsional stiffnesses are derived and shown to be significantly larger than the typically-used *GJ*/*L*. The exact element stiffness matrix associated with the element-end torsion and warping deformations can be obtained, and the exact three-dimensional element stiffness matrix can be further assembled. Some application examples of the exact element stiffness matrix for torsion and warping are also presented.

In addition, the temperature is non-negligible, which can also influence the bifurcation buckling of thin-walled structures, and the thermoelastic analysis of structures has already been presented in many papers. However, this paper is focused on solving the mechanical buckling of thin-walled members. Therefore, we list some papers [24,25] on thermoelastic analysis as a reference instead of presenting further research.

## 2. Equilibrium Analysis

**Figure 3:**
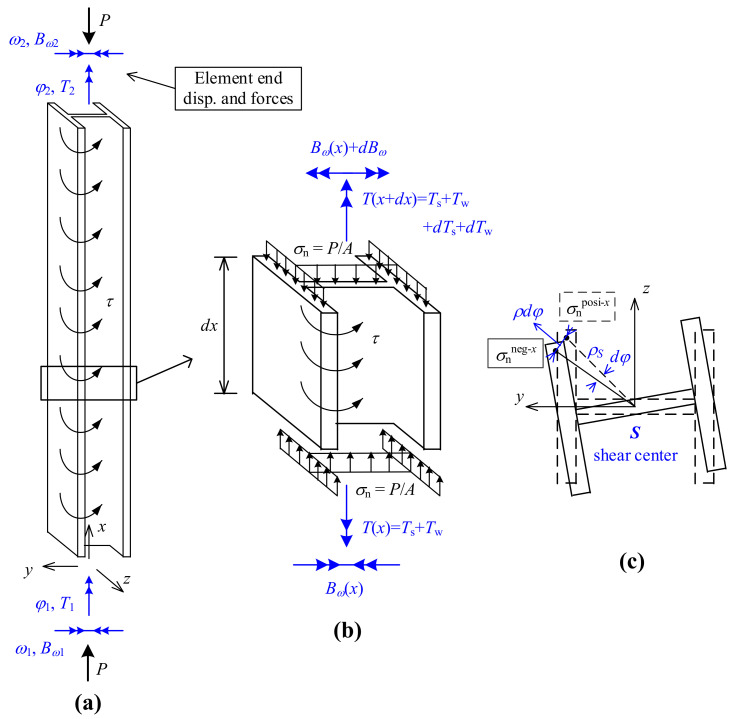
Equilibrium analysis of axial-loaded torsion member: (**a**) analyzed element with element-end torsion/warping deformations and stress resultants; (**b**) equilibrium analysis of element short segment side view; (**c**) cross-section elevation view.

To develop the matrix stiffness method, an equilibrium analysis of an axial-loaded torsion member (especially for members with a thin-walled cross-section) is conducted, as shown in Figure 3.

Besides the usual assumptions of the linear theory of elasticity, the following assumptions [5,6,10,26,27,28] of classical theory for members with a thin-walled cross-section are employed in the analysis:

- The global cross-sectional deformation assumption, i.e., the cross-section is assumed to be perfectly rigid in its own plane while free to warp out of its plane;
- The classical Kirchhoff–Love’s thin plate bending assumption, i.e., straight lines normal to the mid-surface of the thin-walled plates remain straight, inextensible, and normal to the mid-surface after deformation;
- Each thin-walled plate is assumed to have null mid-surface membrane shear strains (Vlasov’s hypothesis) and null transverse extensions.

Based on these assumptions, a compatibility equation relating the angle of twist *φ* and the cross-sectional bimoment *B_ω_* is formulated as Equation (7). The St. Venant torque *T*_s_ is formulated as Equation (8) [5,6,10,26].

(7) $$B_{\omega }=-EI_{\omega \omega }\frac{d^{2}\varphi }{dx^{2}}$$

(8) $$T_{s}=GJ\frac{d\varphi }{dx}$$

where *I_ωω_* denotes the warping constant. The sign conventions of *B_ω_* and *T* are defined in Figure 3b.

Since the total cross-sectional torque *T* is consisted of the *T*_s_ and the warping restraint torque *T*_w_, the *T*_w_ can then be derived as the total cross-sectional torque *T* subtracted by the St. Venant torque

(9) $$T_{w}=T-T_{s}=T-GJ\frac{d\varphi }{dx}$$

1. Equilibrium of torque for element short segment

For an equilibrium analysis, a short segment of the element with length *dx* is analyzed in Figure 3b. The equilibrium of torque gives

(10) $$\frac{dT}{dx}=-\tau$$

where τ denotes the distributed torque.

2. Equilibrium of bimoment for element short segment

The equilibrium of bimoment is also analyzed. The warping restraint torques [Equation (9)] at the two cross-sections with distance x of the element short segment are in the opposite direction, and they combine to a bimoment increment formulated as

(11) $$T_{w}dx=\left(T-GJ\frac{d\varphi }{dx}\right)dx=Tdx-GJd\varphi$$

In addition, due to the torsion of the short segment (cross-section elevation view in Figure 3c), the uniformly distributed axial stress *σ*_n_ on the top section is inclined to produce a shear stress *σ*_n_*dAρ_s_dφ* in the horizontal plane. Therefore, the Wagner effect can be considered by taking moment of the shear stress about the shear center *S*, derived as Equation (12).

(12) $$\int_{A}\left(\sigma_{n}dA\cdot \rho_{S}d\varphi \cdot \rho_{S}\right)=\sigma_{n}d\varphi \int_{A}\rho_{S}{}^{2}dA=\frac{P}{A}I_{p,S}d\varphi$$

where *σ*_n_ = *P*/*A* denotes the assumed uniformly-distributed axial stress from the axial force; *ρ_S_* denotes the distance of a point in the cross-section to the shear center *S*; and *I*_p,*S*_ denotes the polar moment of inertia about the shear center *S*.

Therefore, considering these two effects and an increment of the cross-sectional bimoment along the element length, the equilibrium of bimoment is formulated as Equation (13).

(13) $$Tdx-GJd\varphi +\frac{P}{A}I_{p,S}d\varphi =dB_{\omega }\Rightarrow T=\frac{dB_{\omega }}{dx}-\left(PI_{p,S}/A-GJ\right)\frac{d\varphi }{dx}$$

Then, combining Equations (10) and (13) and considering Equation (7), the governing differential equation of equilibrium for this torsion-warping problem can be established as Equation (14), which can be derived to a dimensionless form as Equation (15).

(14) $$EI_{\omega \omega }\frac{d^{4}\varphi }{dx^{4}}+\left(PI_{p,S}/A-GJ\right)\frac{d^{2}\varphi }{dx^{2}}=\tau$$

(15) $$\begin{array}{cc} \frac{d^{4}\varphi }{dx^{4}}+{\frac{\lambda_{c}}{L^{2}}}^{2}\frac{d^{2}\varphi }{dx^{2}}=\frac{\tau }{EI_{\omega \omega }} & \mathrm{for}\;\;\;PI_{p,S}/A\geq GJ\\ \frac{d^{4}\varphi }{dx^{4}}-{\frac{\lambda_{t}}{L^{2}}}^{2}\frac{d^{2}\varphi }{dx^{2}}=\frac{\tau }{EI_{\omega \omega }} & \mathrm{for}\;\;\;PI_{p,S}/A<GJ \end{array}$$

where *λ*_c/t_ denotes a dimensionless factor for the effect of axial force and St. Venant torsion, defined as Equation (16); the subscripts “c” and “t” are associated with conditions of *PI*_p,*S*_/*A* ≥ *GJ* and *PI*_p,*S*_/*A* < *GJ*, which can be defined as generalized “axial compression” and “axial tensile” situations, respectively.

(16) $$\begin{array}{cc} \lambda_{c}=\sqrt{\frac{PI_{p,S}/A-GJ}{EI_{\omega \omega }/L^{2}}} & \mathrm{for}\;\;\;PI_{p,S}/A\geq GJ\\ \lambda_{t}=\sqrt{\frac{GJ-PI_{p,S}/A}{EI_{\omega \omega }/L^{2}}} & \mathrm{for}\;\;\;PI_{p,S}/A<GJ \end{array}$$

For null distributed torque (τ = 0), the general solution for the differential equation of equilibrium is given by

(17) $$\varphi =\left\{\begin{array}{cc} Q_{1}\cos\left(\lambda_{c}x/L\right)+Q_{2}\sin\left(\lambda_{c}x/L\right)+Q_{3}x+Q_{4} & \mathrm{for}\;\;\;PI_{p,S}/A\geq GJ\\ Q_{1t}\cosh\left(\lambda_{t}x/L\right)+Q_{2t}\sinh\left(\lambda_{t}x/L\right)+Q_{3t}x+Q_{4t} & \mathrm{for}\;\;\;PI_{p,S}/A<GJ \end{array}\right.$$

where *Q*_1_, *Q*_2_, *Q*_3_, and *Q*_4_ (*Q*_1t_, *Q*_2t_, *Q*_3t_, *Q*_4t_) are deformation combination factors defining the possible deformation curve.

In the following, the derivations will focus on the generalized “axial compression” situation. It is noted that the derivations and the results for the generalized “axial tensile” situation is very similar to the “axial compression” situation, and the main difference is the use of hyperbolic trigonometric functions instead of trigonometric functions [as shown in Equation (17)].

Based on Equations (7) and (13), the element stress resultants (bimoment and torque) can be derived as Equations (18) and (19), respectively.

(18) $$\frac{B_{\omega }}{EI_{\omega \omega }}=-\frac{d^{2}\varphi }{dx^{2}}=Q_{1}{\left(\frac{\lambda_{c}}{L}\right)}^{2}\cos\frac{\lambda_{c}x}{L}+Q_{2}{\left(\frac{\lambda_{c}}{L}\right)}^{2}\sin\frac{\lambda_{c}x}{L}$$

(19) $$\frac{T}{EI_{\omega \omega }}=\frac{d\left(B_{\omega }/EI_{\omega \omega }\right)}{dx}-{\left(\frac{\lambda_{c}}{L}\right)}^{2}\frac{d\varphi }{dx}=-{\left(\frac{\lambda_{c}}{L}\right)}^{2}Q_{3}$$

Analysis associated with the element deformations and stress resultants of torsion members can then be conducted using Equations (17)–(19).

## 3. Matrix Stiffness Method for Torsion and Warping

An element stiffness matrix, showing the relationship between the element-end deformations (angle and rate of twist) and the corresponding stress resultants (torque and bimoment), is formulated based on the previous section. Then, a matrix stiffness method for torsion and warping is established for the analysis of torsion members, including the approximate element torsion-warping stiffness matrix for simpler applications and the torsional stiffness analysis for three typical element-end warping conditions.

### 3.1. Element Stiffness Matrix for Torsion and Warping

Equations (17)–(19) can be used in the analysis associated with the element deformations and stress resultants of torsion members. In a matrix stiffness method, attentions are focused on the element-end torsion/warping deformations and the corresponding stress resultants, formulated in matrix forms for simplified and systematic analysis.

The element-end deformations (angle and rate of twist) are formulated in a matrix form as Equation (20) based on Equation (17).

(20) $$\left(\begin{array}{c} \varphi_{1}\\ \varphi_{2}\\ \omega_{1}\\ \omega_{2} \end{array}\right)=\left(\begin{array}{c} \varphi (0)\\ \varphi (L)\\ \omega (0)\\ \omega (L) \end{array}\right)=\left(\begin{array}{cccc} 1 & 0 & 0 & 1\\ \cos\lambda_{c} & \sin\lambda_{c} & L & 1\\ 0 & \lambda_{c}/L & 1 & 0\\ -\lambda_{c}\sin\lambda_{c}/L & \lambda_{c}\cos\lambda_{c}/L & 1 & 0 \end{array}\right)\left(\begin{array}{c} Q_{1}\\ Q_{2}\\ Q_{3}\\ Q_{4} \end{array}\right)$$

The element-end stress resultants (torque and bimoment) are formulated in a matrix form as Equation (21) based on Equations (18) and (19).


(21) $$\left(\begin{array}{c} T_{1}\\ T_{2}\\ B_{\omega 1}\\ B_{\omega 2} \end{array}\right)=\left(\begin{array}{c} -T\left(0\right)\\ T\left(L\right)\\ B_{\omega }\left(0\right)\\ -B_{\omega }\left(L\right) \end{array}\right)=EI_{\omega \omega }\left(\begin{array}{cccc} 0 & 0 & {\left(\lambda_{c}/L\right)}^{2} & 0\\ 0 & 0 & -{\left(\lambda_{c}/L\right)}^{2} & 0\\ {\left(\lambda_{c}/L\right)}^{2} & 0 & 0 & 0\\ -{\left(\lambda_{c}/L\right)}^{2}\cos\lambda_{c} & -{\left(\lambda_{c}/L\right)}^{2}\sin\lambda_{c} & 0 & 0 \end{array}\right)\left(\begin{array}{c} Q_{1}\\ Q_{2}\\ Q_{3}\\ Q_{4} \end{array}\right)$$

The relationship between the element-end deformations (angle and rate of twist) and the corresponding stress resultants (torque and bimoment) is then formulated as Equation (22) by combining Equations (20) and (21).

(22) $$\begin{array}{l} \left(\begin{array}{c} T_{1}\\ T_{2}\\ B_{\omega 1}\\ B_{\omega 2} \end{array}\right)=EI_{\omega \omega }\left(\begin{array}{cccc} 0 & 0 & {\left(\lambda_{c}/L\right)}^{2} & 0\\ 0 & 0 & -{\left(\lambda_{c}/L\right)}^{2} & 0\\ {\left(\lambda_{c}/L\right)}^{2} & 0 & 0 & 0\\ -{\left(\lambda_{c}/L\right)}^{2}\cos\lambda_{c} & -{\left(\lambda_{c}/L\right)}^{2}\sin\lambda_{c} & 0 & 0 \end{array}\right)\left(\begin{array}{cccc} \frac{1-\cos\lambda_{c}}{\phi_{c}} & \frac{\sin\lambda_{c}-\lambda_{c}\cos\lambda_{c}}{k\phi_{c}} & -\frac{1-\cos\lambda_{c}}{\phi_{c}} & \frac{\lambda_{c}-\sin\lambda_{c}}{k\phi_{c}}\\ \frac{k\sin\lambda_{c}}{\phi_{c}} & \frac{1-\cos\lambda_{c}}{\phi_{c}} & -\frac{k\sin\lambda_{c}}{\phi_{c}} & \frac{1-\cos\lambda_{c}}{\phi_{c}}\\ -\frac{\sin\lambda_{c}}{\phi_{c}} & \frac{1-\cos\lambda_{c}-\lambda_{c}\sin\lambda_{c}}{k\phi_{c}} & \frac{\sin\lambda_{c}}{\phi_{c}} & -\frac{1-\cos\lambda_{c}}{k\phi_{c}}\\ \frac{1-\cos\lambda_{c}-\lambda_{c}\sin\lambda_{c}}{\phi_{c}} & -\frac{\sin\lambda_{c}-\lambda_{c}\cos\lambda_{c}}{k\phi_{c}} & \frac{1-\cos\lambda_{c}}{\phi_{c}} & -\frac{\lambda_{c}-\sin\lambda_{c}}{k\phi_{c}} \end{array}\right)\left(\begin{array}{c} \varphi_{1}\\ \varphi_{2}\\ \omega_{1}\\ \omega_{2} \end{array}\right)\\ =\left[K_{e}\right]\left(\begin{array}{c} \varphi_{1}\\ \varphi_{2}\\ \omega_{1}\\ \omega_{2} \end{array}\right) \end{array}$$

where [***K*_e_**] is the element stiffness matrix for torsion and warping, which can be simplified as

(23) $$\left[K_{e}\right]=\left(\begin{array}{cccc} T_{c}(\lambda_{c})\frac{EI_{\omega \omega }}{L^{3}} & -T_{c}(\lambda_{c})\frac{EI_{\omega \omega }}{L^{3}} & Q_{c}(\lambda_{c})\frac{EI_{\omega \omega }}{L^{2}} & Q_{c}(\lambda_{c})\frac{EI_{\omega \omega }}{L^{2}}\\ & T_{c}(\lambda_{c})\frac{EI_{\omega \omega }}{L^{3}} & -Q_{c}(\lambda_{c})\frac{EI_{\omega \omega }}{L^{2}} & -Q_{c}(\lambda_{c})\frac{EI_{\omega \omega }}{L^{2}}\\ & & S_{c}(\lambda_{c})\frac{EI_{\omega \omega }}{L} & C_{c}(\lambda_{c})\frac{EI_{\omega \omega }}{L}\\ \mathrm{sym}. & & & S_{c}(\lambda_{c})\frac{EI_{\omega \omega }}{L} \end{array}\right)\;\begin{array}{l} T_{c}\left(\lambda_{c}\right)=\lambda_{c}{}^{3}\sin\lambda_{c}/\phi_{c}\left(\lambda_{c}\right)\\ Q_{c}\left(\lambda_{c}\right)=\lambda_{c}{}^{2}\left(1-\cos\lambda_{c}\right)/\phi_{c}\left(\lambda_{c}\right)\\ S_{c}\left(\lambda_{c}\right)=\lambda_{c}\left(\sin\lambda_{c}-\lambda_{c}\cos\lambda_{c}\right)/\phi_{c}\left(\lambda_{c}\right)\\ C_{c}\left(\lambda_{c}\right)=\lambda_{c}\left(\lambda_{c}-\sin\lambda_{c}\right)/\phi_{c}\left(\lambda_{c}\right)\\ \phi_{c}\left(\lambda_{c}\right)=2-2\cos\lambda_{c}-\lambda_{c}\sin\lambda_{c} \end{array}$$

where the expressions for the element stiffness coefficients *T*_c_, *Q*_c_, *S*_c_, and *C*_c_ are the same as that in the element stiffness matrix for a flexural-axial problem, which are formulated in Equation (4).

Equation (23) gives the element stiffness matrix based on the axial (compression) force factor *λ*_c_ (Equation (16)) in the case of *PI*_p,*S*_/*A* ≥ *GJ*.

In the case of *PI*_p,*S*_/*A* < *GJ*, the element stiffness matrix can also be derived from the general solution in Equation (17) and is then formulated in the same form as Equation (23), with a change of the subscript “c” to “t”. However, the element stiffness coefficients *T*_t_, *Q*_t_, *S*_t_, and *C*_t_ are expressed in a form using hyperbolic trigonometric functions based on the axial (tension) force factor *λ*_t_.

(24) $$\begin{array}{l} T_{t}\left(\lambda_{t}\right)=\lambda_{t}{}^{3}\sinh\lambda_{t}/\phi_{t}\left(\lambda_{t}\right)\\ Q_{t}\left(\lambda_{t}\right)=\lambda_{t}{}^{2}\left(\cosh\lambda_{t}-1\right)/\phi_{t}\left(\lambda_{t}\right)\\ S_{t}\left(\lambda_{t}\right)=\lambda_{t}\left(\lambda_{t}\cosh\lambda_{t}-\sinh\lambda_{t}\right)/\phi_{t}\left(\lambda_{t}\right)\\ C_{t}\left(\lambda_{t}\right)=\lambda_{t}\left(\sinh\lambda_{t}-\lambda_{t}\right)/\phi_{t}\left(\lambda_{t}\right)\\ \phi_{t}\left(\lambda_{t}\right)=2-2\cosh\lambda_{t}+\lambda_{t}\sinh\lambda_{t} \end{array}$$

**Figure 4:**
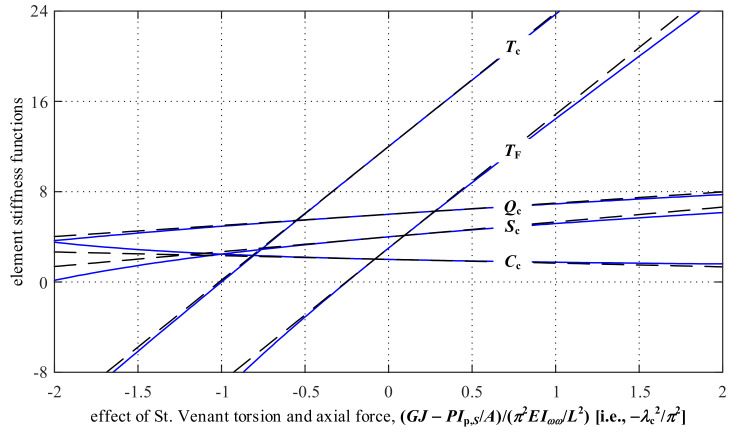
Influence of *GJ*-*PI*_p,S_/*A* on element stiffness functions *T*_c_, *Q*_c_, *S*_c_, *C*_c_, and *T*_F_.

The element stiffness functions *T*_c_, *Q*_c_, *S*_c_, and *C*_c_ correspond to the element-end stress resultant (torque or bimoment) for a unit element-end deformation (angle or rate of twist). These coefficients are transcendental functions of the factor *λ*_c_ for St. Venant torsion and axial force. By noting that $\lambda_{c}=\sqrt{\left(PI_{p,S}/A-GJ\right)/\left(EI_{\omega \omega }/L^{2}\right)}$, the influences of *GJ*−*PI*_p,*S*_/*A* on these element stiffness functions are plotted in Figure 4. As shown in Figure 4, *T*_c_, *Q*_c_, and *S*_c_ increase (while *C*_c_ decreases) with the increase in *GJ*−*PI*_p,*S*_/*A* (which corresponds to either an increasing *GJ* or a decreasing axial force *P*).

The linear approximations of the transcendental element stiffness functions *T*_c_, *Q*_c_, *S*_c_, and *C*_c_ are given as Equation (25) based on the Taylor series of the functions at *λ*_c_ = 0. The approximations are plotted as the dashed lines in Figure 4, which shows that these linear approximations have considerably small differences from the exact transcendental element stiffness functions when *GJ−PI*_p,*S*_/*A* is in a range between −*π*^2^*EI_ωω_*/*L*^2^ and *π*^2^*EI_ωω_*/*L*^2^.

(25) $$\begin{array}{l} T_{c}(\lambda_{c})\approx 12-\frac{6}{5}\lambda_{c}{}^{2}=12+\frac{6}{5}\frac{GJ-PI_{p,S}/A}{EI_{\omega \omega }/L^{2}}\\ Q_{c}(\lambda_{c})\approx 6-\frac{1}{10}\lambda_{c}{}^{2}=6+\frac{1}{10}\frac{GJ-PI_{p,S}/A}{EI_{\omega \omega }/L^{2}}\\ S_{c}(\lambda_{c})\approx 4-\frac{2}{15}\lambda_{c}{}^{2}=4+\frac{2}{15}\frac{GJ-PI_{p,S}/A}{EI_{\omega \omega }/L^{2}}\\ C_{c}(\lambda_{c})\approx 2+\frac{1}{30}\lambda_{c}{}^{2}=2-\frac{1}{30}\frac{GJ-PI_{p,S}/A}{EI_{\omega \omega }/L^{2}} \end{array}$$

Based on Equation (25), the element torsion-warping stiffness matrix [***K*_e_**] can be approximated as Equation (26) for simpler applications, which shows the linear influences of the warping constant *I_ωω_*, the St. Venant torsion constant *J*, and the axial force *P* on [***K*_e_**]. This approximated element stiffness matrix can also be derived by using an energy approach and assuming a cubic deformation shape function [11].

(26) $$\left[K_{e}\right]=\frac{EI_{\omega \omega }}{L^{3}}\left(\begin{array}{cccc} 12 & -12 & 6L & 6L\\ & 12 & -6L & -6L\\ & & 4L^{2} & 2L^{2}\\ \mathrm{sym}. & & & 4L^{2} \end{array}\right)+\frac{GJ-PI_{p,S}/A}{L}\left(\begin{array}{cccc} \frac{6}{5} & -\frac{6}{5} & \frac{L}{10} & \frac{L}{10}\\ & \frac{6}{5} & -\frac{L}{10} & -\frac{L}{10}\\ & & \frac{2L^{2}}{15} & -\frac{L^{2}}{30}\\ \mathrm{sym}. & & & \frac{2L^{2}}{15} \end{array}\right)$$

### 3.2. Torsional Stiffnesses for Three Typical Element-End Warping Conditions

In engineering practices, the element torsional stiffness has drawn most of the attentions, but the warping stiffness and the influence of element-end warping restraint on the torsional stiffness have not been sufficiently investigated.

The element torsional stiffness is usually expressed using the St. Venant torsion constant as *GJ*/*L*. However, because of the interaction between torsion and warping, the element-end torsion stiffness may vary for different warping conditions. Therefore, an element-end torsional stiffness matrix considering different warping conditions is required.

By using the element torsion-warping stiffness matrix [***K*_e_**], we can derive the torsional stiffnesses of members with typical element-end warping conditions.

For members with restrained warping at the ends ($\omega_{1}=\omega_{2}=0$), the rows and columns in [***K*_e_**] related to the rate of twist and bimoment can be deleted to obtain a torsion stiffness matrix.

(27) $$\left[K_{e,\mathrm{tor}}\right]=T_{c}(\lambda_{c})\frac{EI_{\omega \omega }}{L^{3}}\left[\begin{array}{cc} 1 & -1\\ -1 & 1 \end{array}\right]$$

where the torsional stiffness *k*_tor_ for this restrained-restrained warping condition can be expressed as

(28) $$k_{\mathrm{tor}}=T_{c}(\lambda_{c})\frac{EI_{\omega \omega }}{L^{3}}$$

For members with no restraint of warping at the ends (*B_ω_*_1_ = *B_ω_*_2_ =0), the torsion stiffness matrix that relates the element-end twisting angles and torques can be derived as follows.


(29) $$\begin{array}{l} \left(\begin{array}{cc} T_{c}(\lambda_{c})\frac{EI_{\omega \omega }}{L^{3}}\left[\begin{array}{cc} 1 & -1\\ -1 & 1 \end{array}\right] & Q_{c}(\lambda_{c})\frac{EI_{\omega \omega }}{L^{2}}\left[\begin{array}{cc} 1 & 1\\ -1 & -1 \end{array}\right]\\ \mathrm{sym}. & \frac{EI_{\omega \omega }}{L}\left[\begin{array}{cc} S_{c}(\lambda_{c}) & C_{c}(\lambda_{c})\\ C_{c}(\lambda_{c}) & S_{c}(\lambda_{c}) \end{array}\right] \end{array}\right)\left(\begin{array}{c} \left(\begin{array}{l} \varphi_{1}\\ \varphi_{2} \end{array}\right)\\ \left(\begin{array}{l} \omega_{1}\\ \omega_{2} \end{array}\right) \end{array}\right)=\left(\begin{array}{c} \left(\begin{array}{l} T_{1}\\ T_{2} \end{array}\right)\\ \left(\begin{array}{l} 0\\ 0 \end{array}\right) \end{array}\right)\\ \Rightarrow \left\{T_{c}(\lambda_{c})\frac{EI_{\omega \omega }}{L^{3}}\left[\begin{array}{cc} 1 & -1\\ -1 & 1 \end{array}\right]-{\left[Q_{c}(\lambda_{c})\right]}^{2}\frac{EI_{\omega \omega }}{L^{3}}\left[\begin{array}{cc} 1 & 1\\ -1 & -1 \end{array}\right]{\left[\begin{array}{cc} S_{c}(\lambda_{c}) & C_{c}(\lambda_{c})\\ C_{c}(\lambda_{c}) & S_{c}(\lambda_{c}) \end{array}\right]}^{-1}{\left[\begin{array}{cc} 1 & 1\\ -1 & -1 \end{array}\right]}^{T}\right\}\left(\begin{array}{l} \varphi_{1}\\ \varphi_{2} \end{array}\right)=\left(\begin{array}{l} T_{1}\\ T_{2} \end{array}\right)\\ \Rightarrow \left(T_{c}(\lambda_{c})-\frac{2{\left[Q_{c}(\lambda_{c})\right]}^{2}}{S_{c}(\lambda_{c})+C_{c}(\lambda_{c})}\right)\frac{EI_{\omega \omega }}{L^{3}}\left[\begin{array}{cc} 1 & -1\\ -1 & 1 \end{array}\right]\left(\begin{array}{l} \varphi_{1}\\ \varphi_{2} \end{array}\right)=\left(\begin{array}{l} T_{1}\\ T_{2} \end{array}\right) \end{array}$$

By using the element stiffness functions in Equation (23), the torsional stiffness *k*_tor_ for this free-free warping condition is derived as

(30) $$k_{\mathrm{tor}}=\left(T_{c}(\lambda_{c})-\frac{2{\left[Q_{c}(\lambda_{c})\right]}^{2}}{S_{c}(\lambda_{c})+C_{c}(\lambda_{c})}\right)\frac{EI_{\omega \omega }}{L^{3}}=-\lambda_{c}{}^{2}\frac{EI_{\omega \omega }}{L^{3}}=\frac{GJ}{L}-\frac{PI_{p,S}}{AL}$$

For members with restrained warping at one end ($\omega_{1}=0$) and free warping at the other end (*B_ω_*_2_ = 0), the torsion stiffness matrix can be derived as follows.

(31) $$\begin{array}{l} \left(\begin{array}{ccc} T_{c}(\lambda_{c})\frac{EI_{\omega \omega }}{L^{3}} & -T_{c}(\lambda_{c})\frac{EI_{\omega \omega }}{L^{3}} & Q_{c}(\lambda_{c})\frac{EI_{\omega \omega }}{L^{2}}\\ & T_{c}(\lambda_{c})\frac{EI_{\omega \omega }}{L^{3}} & -Q_{c}(\lambda_{c})\frac{EI_{\omega \omega }}{L^{2}}\\ \mathrm{sym}. & & S_{c}(\lambda_{c})\frac{EI_{\omega \omega }}{L} \end{array}\right)\left(\begin{array}{c} \varphi_{1}\\ \varphi_{2}\\ \omega_{2} \end{array}\right)=\left(\begin{array}{c} T_{1}\\ T_{2}\\ B_{\omega 2}=0 \end{array}\right)\\ \Rightarrow \left\{T_{c}(\lambda_{c})\frac{EI_{\omega \omega }}{L^{3}}\left[\begin{array}{cc} 1 & -1\\ -1 & 1 \end{array}\right]-{\left[Q_{c}(\lambda_{c})\right]}^{2}\frac{EI_{\omega \omega }}{L^{3}}\left[\begin{array}{c} 1\\ -1 \end{array}\right]{\left[S_{c}(\lambda_{c})\right]}^{-1}{\left[\begin{array}{c} 1\\ -1 \end{array}\right]}^{T}\right\}\left(\begin{array}{l} \varphi_{1}\\ \varphi_{2} \end{array}\right)=\left(\begin{array}{l} T_{1}\\ T_{2} \end{array}\right)\\ \Rightarrow \left(T_{c}(\lambda_{c})-\frac{{\left[Q_{c}(\lambda_{c})\right]}^{2}}{S_{c}(\lambda_{c})}\right)\frac{EI_{\omega \omega }}{L^{3}}\left[\begin{array}{cc} 1 & -1\\ -1 & 1 \end{array}\right]\left(\begin{array}{l} \varphi_{1}\\ \varphi_{2} \end{array}\right)=\left(\begin{array}{l} T_{1}\\ T_{2} \end{array}\right) \end{array}$$

By using the element stiffness functions in Equation (23), the torsional stiffness *k*_tor_ for this restrained-free warping condition is derived as

(32) $$k_{\mathrm{tor}}=T_{F}(\lambda_{c})\frac{EI_{\omega \omega }}{L^{3}}=\left(T_{c}(\lambda_{c})-\frac{{\left[Q_{c}(\lambda_{c})\right]}^{2}}{S_{c}(\lambda_{c})}\right)\frac{EI_{\omega \omega }}{L^{3}}=\frac{-\lambda_{c}{}^{2}}{1-\tan\lambda_{c}/\lambda_{c}}\frac{EI_{\omega \omega }}{L^{3}}$$

where *T*_F_ is a stiffness function associated with this restrained-free-warping condition, and its linear approximation is derived as Equation (33). The stiffness function *T*_F_ and its approximation are also plotted in Figure 4.

(33) $$T_{F}(\lambda_{c})\approx 3+\frac{6}{5}\frac{GJ-PI_{p,S}/A}{EI_{\omega \omega }/L^{2}}$$

**Figure 5:**
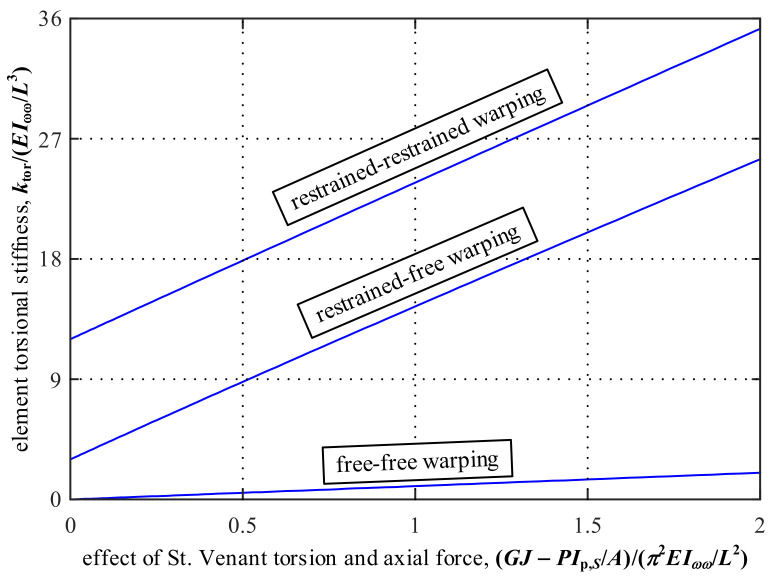
Comparisons of torsional stiffnesses for the three typical warping conditions.

In summary, the torsional stiffnesses of members with three typical element-end warping conditions can be formulated as

(34) $$k_{\mathrm{tor}}=\left\{\begin{array}{cc} T_{c}(\lambda_{c})\frac{EI_{\omega \omega }}{L^{3}}\approx \frac{6}{5}\left(\frac{GJ}{L}-\frac{PI_{p,S}}{AL}\right)+12\frac{EI_{\omega \omega }}{L^{3}} & \mathrm{restrained}-\mathrm{restrained}\;\mathrm{warping}\\ -\lambda_{c}{}^{2}\frac{EI_{\omega \omega }}{L^{3}}=\frac{GJ}{L}-\frac{PI_{p,S}}{AL} & \mathrm{free}-\mathrm{free}\;\mathrm{warping}\\ T_{F}(\lambda_{c})\frac{EI_{\omega \omega }}{L^{3}}\approx \frac{6}{5}\left(\frac{GJ}{L}-\frac{PI_{p,S}}{AL}\right)+3\frac{EI_{\omega \omega }}{L^{3}} & \mathrm{restrained}-\mathrm{free}\;\mathrm{warping} \end{array}\right.$$

where the linear approximations are based on Section 3.1. Equation (34) shows that the commonly-used expression *GJ*/*L* for the torsional stiffness is only valid for members with the free-warping condition and negligible axial force effect. Figure 5 compares the torsional stiffnesses for these three typical warping conditions. The effect of St. Venant torsion and axial force is varied along the horizontal axis. As shown in Figure 5, the torsional stiffness associated with the restrained-restrained warping condition may be significantly larger than the commonly-used value *GJ*/*L*. Therefore, in structural analysis, the torsional stiffness value should be carefully selected based on the element-end warping conditions.

## 4. Applications of Torsion-Warping Stiffness Matrix

In this section, some classical torsion-warping problems will be analyzed as application examples to demonstrate the established matrix stiffness method.

### 4.1. Elastic Buckling Analysis for Typical Torsion-Warping-Axial Stability Problems

The elastic bifurcation buckling analysis can be directly conducted using the assembled structural stiffness matrix [***K*_s_**]. The eigenproblem for an elastic buckling analysis tries to solve a nontrivial solution for the global load deformation equation [***K*_s_**]Δ = 0. Therefore, an elastic buckling analysis can be conducted by setting the determinant of the assembled structural stiffness matrix [***K*_s_**] to zero (Equation (35)). The factor *λ*_c,cr_ at the buckling state can be solved, corresponding to a buckling axial force *P*_cr_ formulated as Equation (36).

(35) $$\det\left[K_{s}(\lambda_{c,\mathrm{cr}})\right]=0$$

(36) $$\sqrt{\frac{P_{\mathrm{cr}}I_{p,S}/A-GJ}{EI_{\omega \omega }/L^{2}}}=\lambda_{c,\mathrm{cr}}\Rightarrow P_{\mathrm{cr}}=\left(GJ+\lambda_{c,\mathrm{cr}}{}^{2}EI_{\omega \omega }/L^{2}\right)A/I_{p,S}$$

For torsion-warping-axial stability problems with typical element-end boundary conditions, the elastic bifurcation buckling analysis can be conveniently conducted using the matrix stiffness method, as listed in Table 1.

For a torsion restrained column (*φ*_1_ = 0, *φ*_2_ = 0) with free of warping at the two element ends (case 1), the unrestrained element-end deformations are the warping deformations. Therefore, the buckling state corresponds to the condition that the determinant of the submatrix for warping (third and fourth row/column of the element stiffness matrix [***K*_e_**]) equals zero, and the buckling condition in this case can be solved as

(37) $$\det\left[\begin{array}{cc} S_{c}(\lambda_{c}) & C_{c}(\lambda_{c})\\ C_{c}(\lambda_{c}) & S_{c}(\lambda_{c}) \end{array}\right]=0\Leftrightarrow S_{c}(\lambda_{c})-C_{c}(\lambda_{c})=0\Rightarrow \lambda_{c,\mathrm{cr}}=\pi$$

For a column with torsion and warping restraints only at one node (*φ*_1_ = 0, $\omega_{1}$= 0) (case 2), the unrestrained element-end deformations are the torsion and warping deformations at the other node. Therefore, the buckling state corresponds to the condition that the determinant of the stiffness matrix at the unrestrained node (second and fourth row/column of the element stiffness matrix [***K*_e_**]) equals zero, and the buckling condition in this case can be solved as

(38) $$\det\left[\begin{array}{cc} T_{c}(\lambda_{c}) & -Q_{c}(\lambda_{c})\\ -Q_{c}(\lambda_{c}) & S_{c}(\lambda_{c}) \end{array}\right]=0\Leftrightarrow T_{c}(\lambda_{c})S_{c}(\lambda_{c})-Q_{c}{\left(\lambda_{c}\right)}^{2}=0\Rightarrow \lambda_{c,\mathrm{cr}}=\pi /2$$

For a column with torsion and warping restraints at one node (*φ*_1_ = 0, $\omega_{1}$ = 0) and a warping restraint at the other node ($\omega_{2}$= 0) (case 3), the only unrestrained element-end deformation is the torsion deformation at the other node. The relationship between this unrestrained torsion deformation and the corresponding element-end torque is defined by *T*_c_(*λ*_c_). Therefore, the buckling state corresponds to the condition that *T*_c_(*λ*_c_) = 0. Based on Figure 4, *T*_c_ decreases to 0 as *λ*_c_ increases to *π*. Therefore, the buckling condition in this case can be solved as

(39) $$T_{c}(\lambda_{c})=0\Rightarrow \lambda_{c,\mathrm{cr}}=\pi$$

For a column with torsion and warping restraints at one node (*φ*_1_ = 0, $\omega_{1}$= 0) and a torsion restraint at the other node (*φ*_2_ = 0) (case 4), the only unrestrained element-end deformation is the warping deformation at the other node. The relationship between this unrestrained warping deformation and the corresponding element-end bimoment can be defined by *S*_c_(*λ*_c_). Therefore, the buckling state is corresponding to the condition that *S*_c_(*λ*_c_) = 0. Based on Figure 4, *S*_c_ decreases to 0 as *λ*_c_ increases to 1.43*π* (*π*/0.7). Therefore, the buckling condition in this case can be solved as

(40) $$S_{c}(\lambda_{c})=0\Rightarrow \lambda_{c,\mathrm{cr}}\approx \pi /0.7$$

In addition, for a column with torsion and warping restraints at both nodes (case 5), the buckling state is corresponding to the condition that the denominator *Φ*_c_ in *T*_c_, *Q*_c_, *S*_c_, and *C*_c_ equals zero [15]. It can be solved that *Φ*_c_ decreases to 0 as *λ*_c_ increases to 2*π*. Therefore, the buckling condition in this case can be solved as

(41) $$\Phi_{c}(\lambda_{c})=0\Rightarrow \lambda_{c,\mathrm{cr}}=2\pi$$

It is noted that this section gives the same results as that from classical analyses of these torsion-warping-axial buckling problems [4,5,7,8], but the matrix analysis procedure is considerably simplified and is more systematic.

### 4.2. Analysis of Torsion and Warping of a Torsion Member with a Midspan Torque

**Figure 6:**
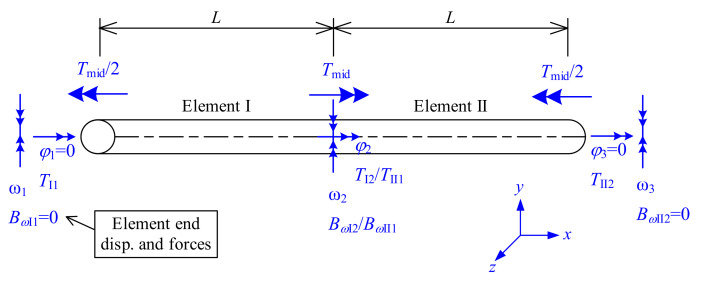
Analysis of torsion and warping of a torsion member with a midspan torque.

This example analyzes a classical torsion-warping problem [4,5], the torsion and warping of a torsion member with a midspan torque, as shown in Figure 6. In nanostructures, torsion members may also be used. Therefore, the matrix stiffness method could be relevant to structural nanomechanics and suitable for nanostructures. In this analysis, the different torque components of the total torque (including the St. Venant torque and the warping restraint torque) are discussed.

The governing equation is formulated as Equation (42), where [***K*_s_**] is the structural stiffness matrix associated with the unconstrained deformations *φ*_2_, $\omega_{1}$, $\omega_{2}$, and $\omega_{3}$. The torsion member is considered to consist of two elements with length *L* (elements I and II in Figure 6). For the two elements, the relationships between their element end deformations (*φ*_1_, *φ*_2_, $\omega_{1}$, and $\omega_{2}$) (or (*φ*_2_, *φ*_3_, $\omega_{2}$, and $\omega_{3}$)) and the corresponding element end stress resultants (*T*_I1_, *T*_I2_, *B_ω_*_I1_, and *B_ω_*_I2_) (or (*T*_II1_, *T*_II2_, *B_ω_*_II1_, and *B_ω_*_II2_)) are both governed by the element stiffness matrix [***K*_e_**] [Equation (23)]. By combining the stability stiffness matrices of element I (row/column 2 to 4 of [***K*_e_**] associated with the unconstrained element end deformations, *φ*_2_, $\omega_{1}$, and $\omega_{2}$) and element II (row/column 1, 3, and 4 of [***K*_e_**] associated with the unconstrained element end deformations, *φ*_2_, $\omega_{2}$, and $\omega_{3}$), the structural stiffness matrix [***K*_s_**] is formulated as Equation (43).

(42) $$\left[K_{s}\right]\left(\begin{array}{c} \varphi_{2}\\ \omega_{1}\\ \omega_{2}\\ \omega_{3} \end{array}\right)=\left(\begin{array}{c} T_{\mathrm{mid}}\\ 0\\ 0\\ 0 \end{array}\right)$$

(43) $$\left.\begin{array}{l} \left[K_{e,I}\right]\left\{2,3,4\right\}\to \left[K_{s}\right]\left\{1,2,3\right\}\\ \left[K_{e,II}\right]\left\{1,3,4\right\}\to \left[K_{s}\right]\left\{1,3,4\right\} \end{array}\right\}\Rightarrow \left[K_{s}\right]=\left(\begin{array}{cccc} 2T_{t}(\lambda_{t})\frac{EI_{\omega \omega }}{L^{3}} & -Q_{t}(\lambda_{t})\frac{EI_{\omega \omega }}{L^{2}} & & Q_{t}(\lambda_{t})\frac{EI_{\omega \omega }}{L^{2}}\\ & S_{t}(\lambda_{t})\frac{EI_{\omega \omega }}{L} & C_{t}(\lambda_{t})\frac{EI_{\omega \omega }}{L} & \\ & & 2S_{t}(\lambda_{t})\frac{EI_{\omega \omega }}{L} & C_{t}(\lambda_{t})\frac{EI_{\omega \omega }}{L}\\ \mathrm{sym}. & & & S_{t}(\lambda_{t})\frac{EI_{\omega \omega }}{L} \end{array}\right)$$

Then, the element-end torsion/warping deformations can be solved based on the relationship in Equation (42). The angle of twist at midspan is

(44) $$\varphi_{2}=\frac{\left(1-\tanh\lambda_{t}/\lambda_{t}\right)T_{\mathrm{mid}}}{2\lambda_{t}{}^{2}EI_{\omega \omega }/L^{3}}$$

For this problem particularly, the element analysis can be conducted for the element I or II. The integration of different torque components (St. Venant torque and warping restraint torque) is analyzed [4,5]. The integration of the St. Venant torque *T*_s_ from the element end to the midspan can be derived (based on Equation (8)) as the product of the St. Venant torsion rigidity *GJ* and the angle of twist at midspan *φ*_2_, as shown in Equation (45). The integration of warping restraint torque *T*_w_ from the element end to the midspan gives the bimoment at midspan *B_ω_*_,mid_ [based on Equation (13)], as shown in Equation (46). The integration of cross-sectional total torque (*T* = *T*_mid_) from the element end to the midspan can be formulated as Equation (47).

(45) $$\int_{0}^{L}T_{s}dx=\int_{0}^{L}\left(GJ\frac{d\varphi }{dx}\right)dx=GJ\varphi_{2}$$

(46) $$\int_{0}^{L}T_{w}dx=\int_{0}^{L}\left(T-T_{s}\right)dx=B_{\omega ,\mathrm{mid}}$$

(47) $$\int_{0}^{L}Tdx=\int_{0}^{L}T_{\mathrm{mid}}/2dx=T_{\mathrm{mid}}L/2$$

**Figure 7:**
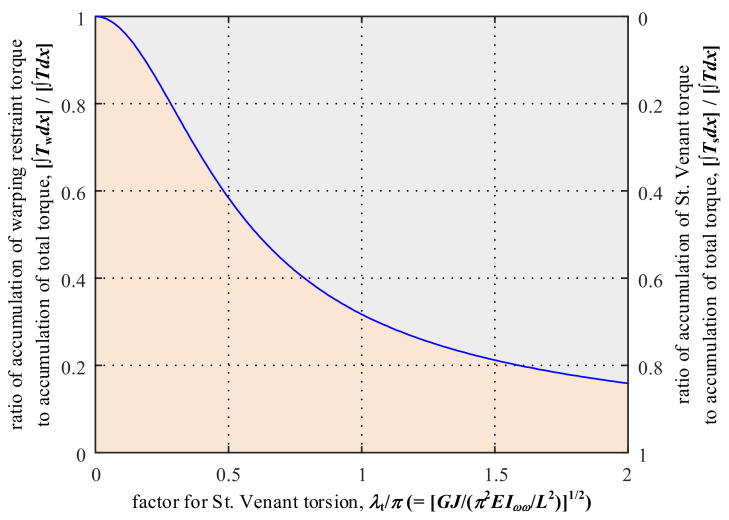
Effect of the factor for St. Venant torsion on the ratio of the accumulation of warping restraint torque/St. Venant torque to the accumulation of total torque.

Then, the ratios of Equation (45) to Equation (47) and Equation (46) to Equation (47) are formulated as Equations (48) and (49), respectively. Equation (48) represents the ratio of the accumulation of St. Venant torque along the element length to the accumulation of total torque, and Equation (49) represents the ratio of the accumulation of warping restraint torque to the accumulation of total torque. The two ratios are plotted in relationships with the factor for St. Venant torsion. As shown in Figure 7, with the increase in the factor for St. Venant torsion (i.e., increase in *GJ*/*EI_ωω_*), the ratio of the accumulation of warping restraint torque decreases, while the ratio of the accumulation of St. Venant torque increases. Therefore, for thin-walled cross-sections with relatively large St. Venant torsion rigidity *GJ* (e.g., closed cross-sections), the St. Venant torque could be dominant in the total torque; in contrast, for cross-sections with relatively small St. Venant torsion rigidity *GJ* (e.g., open cross-sections), the warping restraint torque could be dominant in the total torque.

(48) $$\frac{\int_{0}^{L}T_{s}dx}{\int_{0}^{L}Tdx}=\frac{GJ\varphi_{2}}{T_{\mathrm{mid}}L/2}=\frac{GJ\frac{\left(1-\tanh\lambda_{t}/\lambda_{t}\right)T_{\mathrm{mid}}}{2\lambda_{t}{}^{2}EI_{\omega \omega }/L^{3}}}{T_{\mathrm{mid}}L/2}=1-\tanh\lambda_{t}/\lambda_{t}$$

(49) $$\frac{\int_{0}^{L}T_{w}dx}{\int_{0}^{L}Tdx}=\frac{B_{\omega ,\mathrm{mid}}}{T_{\mathrm{mid}}L/2}=1-\frac{GJ\varphi_{2}}{T_{\mathrm{mid}}L/2}=\tanh\lambda_{t}/\lambda_{t}$$

It is noted that this section gives the same results as that from the analysis in Trahair et al. [4] and Chen [5], but the matrix analysis procedure is considerably simplified and is more systematic.

### 4.3. Second-Order Stiffness Matrix of 3D Beam Considering Exact Torsional Stiffness

Based on the torsion-warping stiffness matrix, this section establishes the element stiffness matrix of axial-loaded three-dimensional beam-columns with a symmetric cross-section.

**Figure 8:**
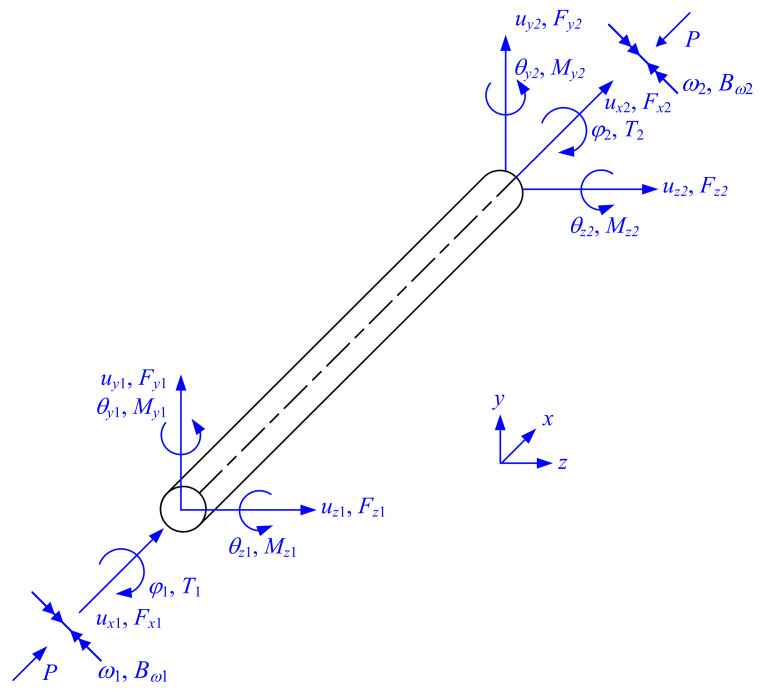
Element-end displacements and forces of 3D beam elements for matrix stiffness method considering warping deformations and bimoments.

The element-end displacement vector of a three-dimensional beam-column with a symmetric cross-section is considered in Equation (50) to include the displacements, the torsional and rotational angles, as well as the rates of twist, as defined in Figure 8.

(50) $$\Delta ={\left(u_{x1},\;u_{y1},\;u_{z1},\;\varphi_{1},\;\theta_{y1},\;\theta_{z1},\;\omega_{1},\;\;u_{x2},\;u_{y2},\;u_{z2},\;\varphi_{2},\;\theta_{y2},\;\theta_{z2},\omega_{2}\right)}^{T}$$

where (*x*, *y*, *z*) denote the local coordinate systems for the three-dimensional element.

The associated element-end stress resultant vector is considered in Equation (51) to include the forces, bending moments and torques, as well as the bimoments.

(51) $$F_{\mathrm{ext}}={\left(F_{x1},\;F_{y1},\;F_{z1},\;T_{1},\;M_{y1},\;M_{z1},\;B_{\omega 1},\;F_{x2},\;F_{y2},\;F_{z2},\;T_{2},\;M_{y2},\;M_{z2},\;B_{\omega 2}\right)}^{T}$$

The element stiffness matrix of three-dimensional beam-columns relates the element-end displacement vector in Equation (50) to the element-end stress resultant vector in Equation (51), and it is, therefore, a 14-degree-of-freedom stiffness matrix. McGuire et al. [11] noted that the difference between the analyses of planar system and three-dimensional system is essentially quantitative. By considering (1) the stiffness matrix [Equation (6)] associated with the member bending in both the *x*-*y* and *x*-*z* planes and (2) the torsion-warping stiffness matrix (Equation (23))established in this paper, the element stiffness matrix of three-dimensional beam-columns can be obtained:


(52) $$\left[\begin{array}{cccccccccccccc} \frac{EA}{L} & & & & & & & -\frac{EA}{L} & & & & & \\ & T_{c}\left(\lambda_{z}\right)\frac{EI_{zz}}{L^{3}} & & & & Q_{c}\left(\lambda_{z}\right)\frac{EI_{zz}}{L^{2}} & & & -T_{c}\left(\lambda_{z}\right)\frac{EI_{zz}}{L^{3}} & & & & Q_{c}\left(\lambda_{z}\right)\frac{EI_{zz}}{L^{2}}\\ & & T_{c}\left(\lambda_{y}\right)\frac{EI_{yy}}{L^{3}} & & -Q_{c}\left(\lambda_{y}\right)\frac{EI_{yy}}{L^{2}} & & & & & -T_{c}\left(\lambda_{y}\right)\frac{EI_{yy}}{L^{3}} & & -Q_{c}\left(\lambda_{y}\right)\frac{EI_{yy}}{L^{2}} & \\ & & & T_{c}(\lambda_{c})\frac{EI_{\omega \omega }}{L^{3}} & & & Q_{c}\left(\lambda_{c}\right)\frac{EI_{\omega \omega }}{L^{3}} & & & & -T_{c}(\lambda_{c})\frac{EI_{\omega \omega }}{L^{3}} & & & Q_{c}\left(\lambda_{c}\right)\frac{EI_{\omega \omega }}{L^{3}}\\ & & & & S_{c}\left(\lambda_{y}\right)\frac{EI_{yy}}{L} & & & & & Q_{c}\left(\lambda_{y}\right)\frac{EI_{yy}}{L^{2}} & & C_{c}\left(\lambda_{y}\right)\frac{EI_{yy}}{L} & \\ & & & & & S_{c}\left(\lambda_{z}\right)\frac{EI_{zz}}{L} & & & -Q_{c}\left(\lambda_{z}\right)\frac{EI_{zz}}{L^{2}} & & & & C_{c}\left(\lambda_{z}\right)\frac{EI_{zz}}{L}\\ & & & & & & S_{c}\left(\lambda_{c}\right)\frac{EI_{\omega \omega }}{L^{3}} & & & & -Q_{c}\left(\lambda_{c}\right)\frac{EI_{\omega \omega }}{L^{3}} & & & C_{c}\left(\lambda_{c}\right)\frac{EI_{\omega \omega }}{L^{3}}\\ & & & & & & & \frac{EA}{L} & & & & & \\ & & & & & & & & T_{c}\left(\lambda_{z}\right)\frac{EI_{zz}}{L^{3}} & & & & -Q_{c}\left(\lambda_{z}\right)\frac{EI_{zz}}{L^{2}}\\ & & & & & & & & & T_{c}\left(\lambda_{y}\right)\frac{EI_{yy}}{L^{3}} & & Q_{c}\left(\lambda_{y}\right)\frac{EI_{yy}}{L^{2}} & \\ & & & & & & & & & & T_{c}(\lambda_{c})\frac{EI_{\omega \omega }}{L^{3}} & & & -Q_{c}\left(\lambda_{c}\right)\frac{EI_{\omega \omega }}{L^{3}}\\ & & & & & & & & & & & S_{c}\left(\lambda_{y}\right)\frac{EI_{yy}}{L} & \\ & \mathrm{sym}. & & & & & & & & & & & S_{c}\left(\lambda_{z}\right)\frac{EI_{zz}}{L}\\ & & & & & & & & & & & & & S_{c}\left(\lambda_{c}\right)\frac{EI_{\omega \omega }}{L^{3}} \end{array}\right]$$

In view of Equation (52), the stiffnesses associated with the y-axis bending, the z-axis bending, and the torsion and warping are uncoupled. Therefore, row/column 2, 6, 8, 12 of Equation (52) represents the z-axis bending of the element, row/column 3, 5, 9, 11 of Equation (52) represents the y-axis bending, and row/column 4, 10, 13, 14 of Equation (52) represents the torsion and warping.

For beam-columns with the three typical element-end warping conditions discussed in Section 3.2, the 14-degree-of-freedom element stiffness matrix of three-dimensional beam-columns can be reduced to a 12-degree-of-freedom element stiffness matrix. This 12-degree-of-freedom element stiffness matrix is more typically used in a systematic analysis of three-dimensional frame systems [11,15,23] because it can be easily transformed to the element global stiffness matrix by using a transformation matrix relating the local and global coordinate systems.

(53) $$\left(\begin{array}{cccccccccccc} \frac{EA}{L} & & & & & & -\frac{EA}{L} & & & & & \\ & T_{c}\left(\lambda_{z}\right)\frac{EI_{zz}}{L^{3}} & & & & Q_{c}\left(\lambda_{z}\right)\frac{EI_{zz}}{L^{2}} & & -T_{c}\left(\lambda_{z}\right)\frac{EI_{zz}}{L^{3}} & & & & Q_{c}\left(\lambda_{z}\right)\frac{EI_{zz}}{L^{2}}\\ & & T_{c}\left(\lambda_{y}\right)\frac{EI_{yy}}{L^{3}} & & -Q_{c}\left(\lambda_{y}\right)\frac{EI_{yy}}{L^{2}} & & & & -T_{c}\left(\lambda_{y}\right)\frac{EI_{yy}}{L^{3}} & & -Q_{c}\left(\lambda_{y}\right)\frac{EI_{yy}}{L^{2}} & \\ & & & k_{tor} & & & & & & -k_{tor} & & \\ & & & & S_{c}\left(\lambda_{y}\right)\frac{EI_{yy}}{L} & & & & Q_{c}\left(\lambda_{y}\right)\frac{EI_{yy}}{L^{2}} & & C_{c}\left(\lambda_{y}\right)\frac{EI_{yy}}{L} & \\ & & & & & S_{c}\left(\lambda_{z}\right)\frac{EI_{zz}}{L} & & -Q_{c}\left(\lambda_{z}\right)\frac{EI_{zz}}{L^{2}} & & & & C_{c}\left(\lambda_{z}\right)\frac{EI_{zz}}{L}\\ & & & & & & \frac{EA}{L} & & & & & \\ & & & & & & & T_{c}\left(\lambda_{z}\right)\frac{EI_{zz}}{L^{3}} & & & & -Q_{c}\left(\lambda_{z}\right)\frac{EI_{zz}}{L^{2}}\\ & & & & & & & & T_{c}\left(\lambda_{y}\right)\frac{EI_{yy}}{L^{3}} & & Q_{c}\left(\lambda_{y}\right)\frac{EI_{yy}}{L^{2}} & \\ & & & & & & & & & k_{tor} & & \\ & \mathrm{sym}. & & & & & & & & & S_{c}\left(\lambda_{y}\right)\frac{EI_{yy}}{L} & \\ & & & & & & & & & & & S_{c}\left(\lambda_{z}\right)\frac{EI_{zz}}{L} \end{array}\right)\left(\begin{array}{c} u_{x1}\\ u_{y1}\\ u_{z1}\\ \varphi_{1}\\ \theta_{y1}\\ \theta_{z1}\\ u_{x2}\\ u_{y2}\\ u_{z2}\\ \varphi_{2}\\ \theta_{y2}\\ \theta_{z2} \end{array}\right)=\left(\begin{array}{c} F_{x1}\\ F_{y1}\\ F_{z1}\\ T_{1}\\ M_{y1}\\ M_{z1}\\ F_{x2}\\ F_{y2}\\ F_{z2}\\ T_{2}\\ M_{y2}\\ M_{z2} \end{array}\right)$$

In view of Equation (53), the torsional stiffness *k*_tor_ in the 4 and 10 rows/columns should be determined using Section 3.2 based on the element-end warping conditions (instead of directly using the value *GJ*/*L*).

This 3D element stiffness matrix can be readily used to solve the exact solutions of the bifurcation buckling problem of 3D frames as well as the out-of-plane buckling of funicular arches.

## 5. Conclusions

This paper presented a matrix stiffness method for the analysis of torsion and warping that is particularly important in beam-columns with torsional deformations. The main works and conclusions are summarized as follows:

1. Equilibrium analysis of an axial-loaded torsion member was conducted based on the equilibrium conditions of torque and bimoment of an element short segment, and a governing differential equation of equilibrium for the angle of twist along the member was established. The solution of the governing differential equation can be used to analyze the element deformations (angle and rate of twist) and stress resultants (torque and bimoment).
2. The exact element stiffness matrix of the torsion member was formulated, showing the relationship between the element-end torsion/warping deformations and the corresponding stress resultants. A dimensionless factor for the effect of St. Venant torsion and axial force was defined. The element stiffness matrix for torsion and warping was readily used for the bifurcation buckling and second-order analysis of axial-loaded torsion members. The exact element second-order stiffness matrix of three-dimensional beam-columns was further assembled.
3. Based on the element torsion-warping stiffness matrix, the exact element torsional stiffnesses considering the interaction of torsion and warping were derived for three typical element-end warping conditions. The commonly-used expression *GJ*/*L* for the torsional stiffness is only valid for members with the free-warping condition and negligible axial force effect. For members with restrained warping at the ends, the torsional stiffness can be significantly larger. For a notable axial force effect, the torsional stiffness may be reduced. Therefore, in the analysis of thin-walled nanostructural structures, the torsional stiffness value should be carefully selected based on the element-end warping conditions and the axial force level.

## Figures and Tables

**Table 1:** Analysis of torsion-warping-axial stability problems with typical element-end boundary conditions.

Case.	Boundary Condition at Node 1	Boundary Condition at Node 2	Condition for Buckling	Dimensionless Factor at Buckling State (λ_c,cr_)	Buckling Axial Force (*P*_cr_)
1	*φ*_1_ = 0	*φ*_2_ = 0	*S*_c_−*C*_c_ = 0	*λ*_c,cr_ = *π*	$P_{\mathrm{cr}}=\left(GJ+\pi^{2}EI_{\omega \omega }/L^{2}\right)A/I_{p,S}$
2	$\varphi_{1}=0,\;\omega_{1}$= 0	/	*T*_c_*S*_c_−*Q*_c_^2^ = 0	*λ*_c,cr_ = *π*/2	$P_{\mathrm{cr}}=\left(GJ+\pi^{2}EI_{\omega \omega }/4L^{2}\right)A/I_{p,S}$
3	$\varphi_{1}=0,\;\omega_{1}$= 0	$\omega_{2}$=0	*T*_c_ = 0	*λ*_c,cr_ = *π*	$P_{\mathrm{cr}}=\left(GJ+\pi^{2}EI_{\omega \omega }/L^{2}\right)A/I_{p,S}$
4	$\varphi_{1}=0,\;\omega_{1}$= 0	*φ*_2_ = 0	*S*_c_ = 0	*λ*_c,cr_ ≈ *π*/0.7	$P_{\mathrm{cr}}\approx \left(GJ+2.05\pi^{2}EI_{\omega \omega }/L^{2}\right)A/I_{p,S}$
5	$\varphi_{1}=0,\;\omega_{1}$= 0	$\varphi_{2}=0,\;\omega_{2}$= 0	*φ*_c_ = 0	*λ*_c,cr_ = 2*π*	$P_{\mathrm{cr}}=\left(GJ+4\pi^{2}EI_{\omega \omega }/L^{2}\right)A/I_{p,S}$

## Data Availability

Data presented in this article is available on request from corresponding author.

## Nomenclature

*A*	Cross-sectional area
*E*	Elastic modulus
*F*_x1/2_	Axial load at the two element ends
*F*_y1/2_, *F*_z1/2_	Load in y-direction and z-directions, respectively
*G*	Shear modulus
*I*	Moment of inertia
*I*_p,S_ = ∫ρ_S_^2^dA	Polar moment of inertia about the shear center
*I*_yy_, *I*_zz_	Moments of inertia about the y-axis and z-axis, respectively
*I*_ωω_	Warping constant
*J*	St. Venant torsion constant
[***K*****_e_**]	Element stiffness matrix
[***K*****_s_**]	Structural stiffness matrix
*k*_tor_	Torsional stiffness
*L*	Element length
*M*_y1/2_, *M*_z1/2_	Bending moments about the y-axis and z-axis, respectively
*P*	Axial compressive force
*P*_cr_	Buckling axial force
*Q*_1_, *Q*_2_, *Q*_3_, *Q*_4_ (*Q*_1t_, *Q*_2t_, *Q*_3t_, *Q*_4t_)	Deformation combination factors defining the possible deformation curve
*T*_c/t_, *Q*_c/t_, S_c/t_, C_c/t_, *T_F_*, φ_c/t_	Coefficients in the element stiffness matrix as functions of λ_c/t_
*T*_mid_	Applied midspan torque
*T*_s_, *T*_w_	St. Venant torque and warping restraint torque, respectively
*T*(x), *B*_ω_(x)	Torque and bimoment at the cross-section considered, respectively
*T*_1/2_, *B*_ω1/2_	Torques and bimoments at the two element ends, respectively
*u*_y1/2_, *u*_z1/2_	Translational displacements in y-direction and z-direction, respectively
Δ, ***F*_ext_**	Element-end deformation vector and corresponding stress resultant vector
*θ*_y1/2_, *θ*_z1/2_	Rotation angles in the X-Z plane and X-Y plane, respectively
λ_c/t_	Factor for the effect of axial force and St. Venant torsion
λ_c,cr_	Factor for axial force and St. Venant torsion at the buckling state
λ_y_, λ_z_	Factors for axial force associated with the y-axis bending and z-axis bending, respectively
*ρ*_S_	Distance of a point in the cross-section to the shear center
σ_n_	Assumed uniformly-distributed axial stress from the axial force
τ	Distributed torque
φ,ω	Angle and rate of twist, respectively
φ_1/2_, ω_1/2_	Angles and rates of twist at the two element ends, respectively
